# Dinamic balance, lifestyle and emotional states in young adults

**DOI:** 10.1590/S1808-86942010000300020

**Published:** 2015-10-20

**Authors:** Ivana Beatrice Manica da Cruz, Daniele Coronel Mena Barreto, Andressa Boer Fronza, Ivo Emilio da Cruz Jung, Cristina Costa Krewer, Maria Izabel de Ugalde Marques da Rocha, Aron Ferreira da Silveira

**Affiliations:** 1 PhD. Post-Doctorate. Adjunct Professor; 2 Speech and Hearing Therapist. MSc student – Graduate Program in Human Communication Disorders; 3 Speech and Hearing Therapist. MSc student – Graduate Program in Human Communication Disorders; 4 Psychology Student, Scientific Initiation; 5 MSc. Assistant Professor; 6 PhD, Adjunct Professor; 7 PhD, Adjunct Professor – Advisor at the Graduate Program in Human Communication Disorders. Federal University of Santa Maria (UFSM) – Morphology Department – Health Sciences Center

**Keywords:** anxiety, smoking, musculoskeletal equilibrium, life style, depression

## Abstract

Aone hypothesis to explain some vestibular peripheral disorders is their association with life style. Thus, studies with young adults are relevant.

**Aim:** to analyze the prevalence of dynamic balance alterations in young adults and their possible association with life style variables, health and negative emotional states (since they can impact the outcome).

**Materials and Methods:** we carried out a non-probabilistic cross-sectional, clinical-retrospective study with young adults (18-32 years of age) from a university with 751 individuals, with a mean age of 22.45± 3.32 years. Life style, health and negative emotional states (NES) variables – depression, stress and anxiety, were collected by means of an interview. The Unterberger test was used in order to check for indications of dynamic balance alterations. Individuals with altered dynamic balance (ADB) were compared to those without these alterations (controls).

**Results:** From our sample, 642 (83.6%) had less than 45°$ of deviation, while 109 (14.2%) had greater than 45° deviation and were the considered with ADB. The ADB group had a greater prevalence of smoking, alcohol abuse/dependence, high blood pressure and NES.

**Conclusion:** the study highlights the occurrence of ADB which needs to be corroborated in future studies.

## INTRODUCTION

Walking is a common movement performed by human beings and it is controlled by the nervous system which regulates the temporal-space association between the position and the movement maintaining body ballance^1^. The signs and symptoms of body balance change can crop up when there is a conflict in the integration of vestibular information, appearing upon rotational dizziness (vertigo) and/or the non-rotational^2^. The physical decline associated with this movement seems to be associated with aging and to non-transmissible chronic diseases^3^, ^4^, ^5^. In this case, changes associated with aging of the psychomotor, sensorimotor and neuromuscular systems cause the appearance of postural balance problems, thus increasing the risk of falls. In Group B, the patients had sensorineural hearing loss in mild to moderate levels, unilaterally in six ^6^,^7^.

The capacity a person has to close the eyes and walk without moving, depends on a normal vestibulo-spinal and proprioceptive functions. With aging, such capacity also suffers changes such as the presence of vestibular dysfunctions as Ménière's disease, central vestibular lesions, multiple sclerosis, etc. Nonetheless, vestibular dysfunctions may also have a vascular lesion associated to the vertebrobasilar system, which is responsible for the cochleovestibular blood irrigation^8^.

Recently, a hypothesis to explain peripheral vestibular disorder of vascular origin would be its correlation with morbidities associated to lifestyle and atherosclerosis. Some authors^9^ consider this hypothesis in their study, using carotid ultrasonography in order to assess the diameter of the intima layer of these vessels and biochemical markers in 85 patients complaining of dizziness. Results describe that there is a positive correlation between peripheral vestibular disorders and vascular changes associated to atherosclerosis. An epidemiological study from the National Health and Nutrition Examination Survey which included 1,685 individuals with age equal to or higher than 50 years, observed a gait problem in women with low levels of HDL-cholesterol and a greater frequency of abdominal obesity^10^. Other studies also describe an association between gait disorders and abdominal obesity^11^,^12^.

Nonetheless, epidemiological studies, especially in young adults who have still not accumulated a large number of dysfunctions and morbidities are insipient and would be relevant to analyze how much vascular change – as is the case of cardiovascular risk factors – could influence body balance.

Within the main factors associated to vascular changes which could affect the vestibular system we have: smoking, systemic hypertension, obesity – for its association with atherosclerotic, inflammatory changes and diabetes mellitus^13^,^14^.

Given the relevance of the topic and the low quality of studies associating balance changes and (cardio) vascular risks, we led a cross-sectional populational study in young adults in whom we noticed the prevalence of individuals with dynamic balance disorders using the Unterberger test and the following risk factors: smoking, overweight/obesity, a past of hypertension and diabetes mellitus. Since there may be changes in dynamic balance associated to emotional states, we also investigated if individuals with indication of dynamic balance changes could have a greater prevalence of self-reported negative emotional states (NES) (anxiety, stress and depression).

## MATERIALS AND METHODS

### Outlining, population and sample

We did a cross-sectional epidemiological study in young adults who were previously selected for studies on environmental-genetic factors associated with smoking and other cardiovascular risks. Our study sample was made up of a university community. The sample selection was non-probabilistic, done from a complementary undergraduate course aimed at epidemiological investigations of aging, which included 122 undergraduate students from courses in the fields of health and biology. These students were trained as research assistants as to methodological, ethical, structured interview deployment and Unterberger balance test aspects. Each student invited, read and collected the patient's signature in the informed consent form (TCLE) and collected information from 10 volunteers (05 men and 05 women) with ages between 18 and 32 years. Once the information was collected, they were plotted by the research assistant. Afterwards, data truthfulness was checked as well as information collection quality and data typing in an Excel spreadsheet by a team of undergraduates and graduate students (masters and doctorates) who participated in the study. The calculation of sample size estimated the inclusion of a minimum number of 600 and a maximum of 1200 individuals. A total of 1,024 individuals with mean ages of 22.45± 3.32 (18 to 32 years) were included in the study. From these, we selected 751 individuals for this study for presenting similar cultural and socio-economic characteristics. The project which this study was part of was previously approved by the Ethics in Research Committee of this Institution, under process # 23081.012293/2007-03. All the volunteers signed the TCLE and the study was led within the standards of resolution 196/1996 from the National Council of Ethics in Research (CONEP).

### Variables studied

The general data on socio-economic, cultural, lifestyle and health indicators were collected through a structured interview. The average time of questionnaire application was 20-25 minutes. Anthropometric data on weight (Kg) and height (m) was used to calculate the body mass index (BMI, Kg/m2) which later served to classify the individuals as non-obese (BMI < 25 kg/m2), overweight (>25<30 Kg/m2) and obese (>30 kg/m2). The waist line (WL) was measured by the research assistants, previously trained to collect such information. The WL was measured at the navel height15. Because of the lack of universally accepted cutting points for WL, we chose to employ the one used in an investigation about the association between WL and health risks (normal for women; < 88 cm and high: > 88cm; normal for men <102 cm, and high > 102 cm)^16^.

Three main lifestyle variables were investigated: smoking, abuse indication and/or alcohol addiction and regular physical activity. Were considered smokers those who reported having smoked at least 100 cigarettes in the past ninety days. The degree of nicotine addiction was also characterized through the Fagerstrom test, which score determines the following addiction categories in points: 0 – 2: very low; 3 – 4: low; 5: mean; 6 – 7: high and 8 −10: very high)^17^. The abuse/addiction concerning alcohol was tested through the CAGE test which is made up of four questions. The affirmative answer for two questions indicated addiction and/or abuse of alcoholic beverages. This score is broadly used by the World Health Organization (WHO)^18^.

Regular physical activity was evaluated through two general questions: whether the volunteer had performed some type of regular physical activity at least twice a week in the past six months. To those individuals who reported some kind of regular physical activity we asked about the type of activity. The other question considered whether the individual, in his daily working activities (studying/ working) remained seated for most of the time. The mean time spent on these activities was also asked.

Negative emotional states were evaluated by means of three general questions whether in the past six months: “the individual felt sad or depressed without apparent cause for at least three times per week “; “felt anxious without apparent cause, for at least three times per week “ and “felt stressed, without apparent cause, for at least three times per week “. We also asked about history of morbidities, especially blood hypertension, diabetes mellitus and dyslipidemias, which are metabolic disorders considered of cardiovascular risk.

In order to assess dynamic balance we used the Unterberger test. We asked our volunteers to walk raising their knees to approximately 45° without actually moving, doing 50 steps (one per second) with their arms extended and eyes closed. We considered altered those individuals who shifted to the right or to the left in 45° or more. All the researchers were instructed to employ this test using a physical therapist or speech and hearing therapist. The test was run in a dark place, with low noise in order to avoid possible interference to the data collected.

In order to do the statistical analysis, we first plotted the data in an Excel spreadsheet and, later, it was transferred and analyzed by the SPSS version 12.0 statistical package. The results obtained were described as mean+standard deviation (SD) or as absolute and relative (%) frequencies according to the type of variable. After estimating the prevalence of individuals with dynamic balance alteration indicated by the test employed (hereby called group with altered dynamic balance, ADB) and the individuals who executed the test within the expected standards (here we called it Control Group) among lifestyle, health and negative emotional status variables, especially those associated with vascular risk. Categorical variables were statistically compared by the Chi-Square or Fisher's exact tests. Continuous variables were compared by the t-student test since these variables have a normal distribution assessed by the Kolmogorov-Smirnof test. The tests in which the significance value was p<0.05 were deemed significant. The multivariate analysis used logistic regression (Backward Wald method) using all the variables associated with dynamic balance change in which the univaried statistical analysis showed a significance value of p<0.1.

## RESULTS

Of the 751 young adults who participated in the study, 642 (83.6%) had less than 45° of sideways shifting in the gait test, while 109 (14.2%) had a shifting greater than 45°, then being considered ADB. In this case, 322 (8.9%) from the control group were women and 320 (85.1%) were men. The number of women with gait deviation was 53 (14.15%) and that of men was 56 (14.9%) and there were no statistically significant differences between the genders (c2=0.087, p=0.773). In the studied sample, 49.1% shifted to the right and 42.5% shifted to the left.

The comparison between the general characteristics of these two groups is summarized on Table 1. As we can see both in men and women, the mean age and the anthropometric variables were statistically similar between the control and the ADB groups.

**Table 1:** General characteristics of young adults without a prior history of vestibular disorders with altered dynamic balance (ADB) and without changes in dynamic balance (control) assessed by the Unterberger test.

Variables		Men			Women	
	Control Mean+SD	ADB Mean+SD	p	Control Mean+SD	ADB Mean+SD	p
Age (years)	22,6±3,2	22,9±2,9	0,447	22,3±3,4	22,6±3,5	0,476
Weight (Kg)	74,6±11,7	76,8±12,6	0,228	59,2±9,9	57,7±7,5	0,304
BMI (Kg/m2)	23,7±3,2	24,3±3,8	0,205	21,7±3,2	21,6±2,9	0,868
Waist (cm)	83,7±9,7	86,4±11,8	0,06	75,1±10,4	75,4±10,5	0,822

ADB= Altered Dynamic Balance; SD= standard deviation; Statistical comparisons were done using the t-Student test.

**Figure 1:**
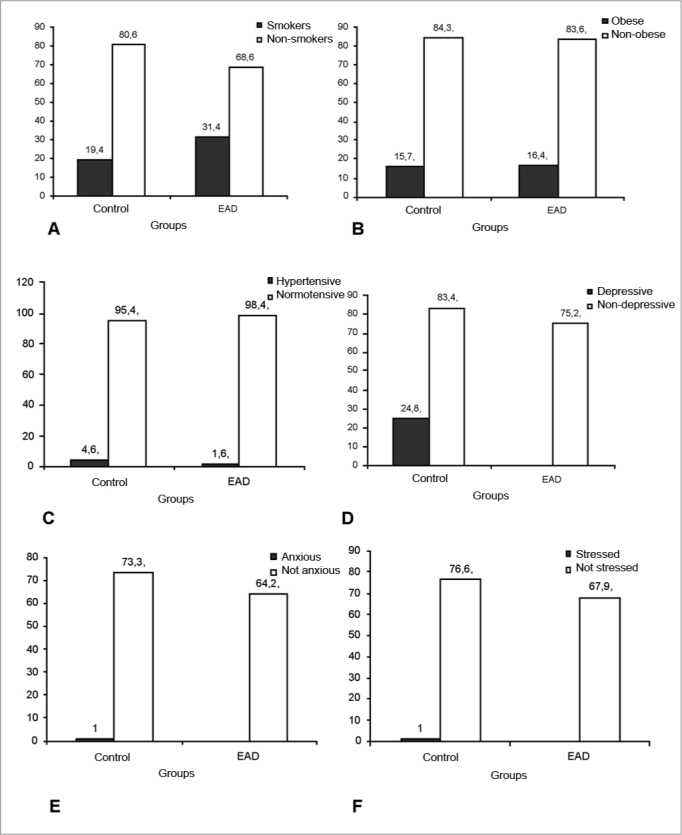
. Comparing the distribution of the frequencies of smoking (A), obesity (B), systemic arterial hypertension (C), self-report of depression (D), anxiety self-report (E) and stress self-report (F) among young adults with ADB and without alteration (controls). – ADB= Altered Dynamic Balance.

Fig. 1 shows the comparison of the cardiovascular risk factors distribution between the control and the ADB groups. In the ADB group we noticed a significantly higher participation of smokers when compared to non-smokers (c2=7.766, p=0.005) with an odds ratio of 1.902 (1.204-3.004) times higher chance of smoking individuals having balance disorders (in other words, being included in the ADB) when compared with non-smokers. The nicotine addiction analyses done by the Fargerstron test showed that, in average, 60.8% (n=73) of smokers had low addiction and 39.2% (n=47) had moderate addiction to nicotine. This ratio was statistically similar in the two groups tested.

The indication of alcohol abuse or addiction evaluated between the two groups showed a higher prevalence of such condition in the ADB group (20.2%, n=22) when compared to the control group (11.9%, n=76). (c2=5.687, p=0.017). The odds ratio of an individual with alcohol abuse/addiction indication to have balance disorders was estimated in OR=1.880 (1.112-3.179).

Another lifestyle variable investigated was associated with physical activity. When the individual was asked whether or not he/she had some physical activity 13.0% (n=50) of those in the ADB group reported having participated in regular physical activity at least twice a week in the past six months, while this frequency in the control group was of 15.8% (n=59) – and these differences were not statistically significant. The most frequent types of physical activities were: aerobic activity (31.0%), weight lifting (24.0%) and some kind of sport (20.7%). Among volunteers, 568 (75.8%) reported doing most of their work/ study seating down, and such ratio was similar between the Control Group and the ADB Group with a mean value of 6.48±2.86 daily hours spent in these activities. This activity was also similar in the Control and ADB groups, hereby investigated.

A total of 117 (16.3%) patients in the sample were classified as being obese, and there were no statistically significant differences in this distribution between young adults from the Control Group and those from the ADB Group (c2=0.030, p=0.863).

Although this is a sample of young adults, 2.0 % (n=15) already had high blood pressure. In this case, ADB individuals had a rate of 4.6% (n=5) of hypertension, while those in the control group had only 1.6% (n=10). In this case, these differences were statistically significant (c2=4.369, p=0.037). The odds ratio of hypertensive individuals having dynamic balance disorders was: OR=3.038 (1.018-9.068) times higher when compared to those with normal blood pressure.

Only three individuals were diabetic, thus it was not possible to do a consistent statistical analysis. Nonetheless, it is worth mentioning that all of them had more than 45° of shifting and were classified in the ADB Group. A total of 29 (3.9%) individuals reported having dyslipidemias, and such disorder did not have a differentiated distribution between Groups Control and ADB (c2=0.175 p=0.675).

Of the young adults investigated, 10 (1.3%) reported having very bad hearing, 11 (1.5%) had a constant tinnitus in one of the ears and 131 (17.4%) reported having tinnitus in certain situations. In this case, none of these conditions was statistically significant when we compared the two groups – Control and ADB. The visual acuity self-perception was also not associated with the altered dynamic balance, happening to 8.7% (n=67) of the individuals who reported having bad or very bad vision.

The ADB group had a significantly higher rate of all the NES indicators (anxiety, depression and stress) as it can be seen in Fig. 1. The odds ratio of individuals with depression having altered dynamic balance was OR=1.653 (1.020-2.677), while individuals with anxiety had an odds ratio of OR=1.531 (1.011-2.352), and stressed individuals had an odds ratio of OR= 1.548 (0.995-2.408).

The multivariate analysis showed that smoking and the negative emotional states (NES) (especially stress) were associated to the dynamic balance of the young adults who were investigated, regardless of gender, age and alcohol abuse/addiction.

## DISCUSSION

The present study evaluated the prevalence of dynamic balance disorders in young adults and its possible association with lifestyle and morbidity variables which are considered cardiovascular risks, in which there was an independent association of smoking and negative emotional states – especially stress.

The results showed a relatively high prevalence of individuals with altered dynamic balance if we consider that here we had a potentially healthy sample. Unfortunately, we did not find a similar populational study which could be used as a reference to compare this prevalence we observed. Despite the few studies in the literature, it is important to mention that the prevalence of individuals with changes in their stationary gait tends to increase very much with aging. According to some authors,19 about 85% of the elderly above 65 years of age complained of postural balance disorders and have, as a consequence, gait shifting, falls and postural instability. Thus, it is considered that such changes arise from organic alterations associated with biological aging. Such changes can be worsening by the presence of chronic non-transmissible diseases such as diabetes mellitus, neurologic diseases, atherosclerosis, depression, and others. In this study involving young adults one can not rule out the fact that the gait disorders seen in the individuals would not be indicators of biological aging of the vestibular system increased by risk factors. Thus, we suggest the need for further epidemiological studies in order to see how much the results obtained come close to the epidemiological reality of the population investigated.

Moreover, we noticed in the sample studied, an almost equal distribution in terms of gait shifting to the right or to the left. The gait deviation without the aid of vision is considered an example of what is called “directional tendency”. These are reported when the person tends to shift to one direction. As far as the static gait is concerned, some studies indicate that two-thirds of the individuals who performed this movement without looking tended to shift to the right^20^, ^21^, ^22^, ^23^. Others did not report a predominant trend^24^while other studies reported a trend of gait shifting to the left^25^. In the present study there was a mild trend towards shifting to the right. These authors^25^ investigated this issue more in depth and suggested that this trend could be associated with factors such as the presence of asymmetry in the mental space representation, vestibular disorder associated with sensory-motor asymmetries or asymmetries in the dopaminergic system. Despite not being the main scope of the present paper and the limitations inherent to general screening studies, it is important to mention that the asymmetry found confirms prior studies that the static gait without vision input is a directional tendency and that we need further studies in order to better understand its mechanisms^25^.

The associations seen between dynamic balance changes and smoking, drinking, hypertension and negative emotional states, even considering that epidemiological studies on this topic are extremely insipient, seems to have a consistent biological support.

Considering the result observed between smoking and stationary gait changes we can comment that smoking addiction is considered one of the best predictors of negative longevity for humans for being associated with an increase in the risk of developing non-transmissible chronic disorders such as cardiovascular, pulmonary and neoplastic diseases. The total number of deaths associated with smoking has reached the figure of 4.9 millions annually, corresponding to more than 10 thousand per day. According to the WHO, should its current expansion trends continue, these figures will increase to 10 million annual deaths around the year of 2030, and half of them will happen to individuals in a productive age (between 35 and 69 years)^26^.

It seems that the negative pleiotropic have an effect on many organs and systems of the body through the presence of a huge number of bioactive substances on which nicotine has a relevant impact. Nicotine, the main active component of tobacco has a high addictive potential, comparable to cocaine and heroin. This substance is quickly absorbed by the central nervous system (CNS). There, it binds to central and peripheral receptors, crossing the blood-brain barrier (nicotinic cholinergic receptors -nAchR) and, thus, increasing the production of neuromediators such as noradrenalin, acetylcholine, serotonin and dopamine^27^.

Even if in a small number, some studies suggest that nicotine can induce an unbalance in the vestibule-ocular and vestibulo-spinal reflexes^28^,^29^. Unfortunately, epidemiological investigations on such associations are very insipient.

Another possible nicotine influence is on modulating the peripheral vasoconstriction of the vestibulo-spinal system. A recent study assessed the effect of vascular risk factors, alone or in combination, on the balance of elderly citizens using a posturography platform. Among the vascular risks tested, glucose intolerance was the only risk factor independently associated with unbalance. Nonetheless, the joint presence of many risk factors, including smoking, seems to cause balance disorders. The authors also comment that such effect seems to be subclinical, associated with nervous system problems impairing body balance30. Therefore, there are indications that smokers could have a higher risk of having dynamic balance disorders even when young. However, such hypothesis needs to be confirmed in future studies which assess other elements associated with body balance.

Negative emotional states were the other set of independent variables associated with dynamic balance changes in the sample of young adults here investigated. Specifically speaking, depression is considered a mood state characterized by feelings of sadness, hopelessness and disheartening. Such state can vary from feelings known as negative emotional states all the way to pathological states such as depression major. In the first case, one can say that there is a state of “reactive depression” associated to the stressful situations of daily life. Prior studies about depression have found an overlapping between the psychiatric and neurological activities. One example of this statement comes from a study^31^ in which 134 depressive patients in whom important neurotological alterations were seen, and also many patients who reported signs of vertigo and nausea, usually found in vestibular disorders. Another investigation which assessed the vestibular system integrity upon depression is the study of the vestibulo-ocular reflex. The ocular reflex movement depends on the vestibular nucleus activity and other authors reported that in depressed patients there was an asymmetry in the reflex stimulus in which the activity detected on the left side was approximately half of the activity observed on the right side. The authors also found a hypoactivity of the vestibular nucleus in individuals with depression when compared to controls, specifically on the right side of the nucleus. These results corroborate through neurophysiology the association between these depressive states (especially in the case of depression major) and balance changes through activity asymmetries in the vestibular nucleus^32^. Here it is interesting to notice that few studies have been done on the static and dynamic balance in young adults and its possible association with negative emotional states or even with the prevalence of neuro-psychiatric disorders.

And finally, it is important to discuss the main limitations of the study hereby described. Investigations on the health indicators or balance changes in large populational samples meddle in the need to apply instruments, which are, in principle, of low cost and easy applicability. One of these tests is commonly used in the clinical evaluation of dynamic balance disorders, which was concocted by Unterberger and later modified by Fukuda^33^ employed here in this investigation. This test has been used as an indicator of vestibular disorder even when there is criticism concerning its real specificity and sensitivity in detecting peripheral vestibular asymmetry in patients affected with this disorder^34^ and the results must be interpreted with caution when the test is used in the diagnosis of these dysfunctions^35^.

One study which compared 21 asymptomatic volunteers with 38 patients with peripheral vestibular disorder, in which the Unterberger-Fukuda test was applied, using data digital capture and analysis, showed that the lateral movement, the pace of the steps, the shoulder shifting was equal in both groups investigated, however the time taken in each step and the angular deviation was greater in the group of patients when compared to the controls^36^. Despite the test limitation concerning its sensitivity and specificity to vestibular disorders, such test has been used in populations without disorders with a relative frequency. Additionally, dynamic balance tests, such as the Unterberger, have been associated with advanced technological equipment which allow for a more accurate monitoring and a greater objective interpretation. This is the case in the study^25^ which quantified the lateral deviation based on the Unterberger-Fukuda test and determined the possible factors which could be potentially associated to these deviations assessing 25 young adults in experimental conditions. Thus, complementary investigations with this technological support could be led with the aim of confirming the associations between dynamic balance, smoking and negative emotional state.

Another limitation of this study is associated with the fact that it is a cross-sectional study and the use of only one test of dynamic balance indication, while usually in the diagnosis of different vestibular disorders numerous tests are applied to the patients. Since this is a small epidemiological initial screening test, such limitation can be corrected in complementary studies about the health of the vestibular-spinal system, especially concerning body balance.

## CONCLUSION

Regardless of methodological limitations, the study hereby reported stresses the occurrence of dynamic balance changes in apparently healthy young adults in the population and that of smoking and negative emotional states as independent risk factors. Such results may be proven through future clinical-epidemiological investigations.
